# The level of skills involved in an observation-based gait analysis

**DOI:** 10.16910/jemr.17.3.1

**Published:** 2024-09-25

**Authors:** Shuzo Bonkohara

**Affiliations:** Department of Physical Therapy, Faculty of Health Science, SBC Tokyo Medical University, Chiba, Japan

**Keywords:** Eye movement, observation, eye tracking, gait analysis, gaze, experience, individual differences

## Abstract

This study aimed to determine the visual assessment skills during an observation-based gait analysis. Participants (N=40) included 20 physiotherapists (PTs) with>10 years of clinical experience (physiotherapists) and 20 physiotherapy students. Both groups watched a video of the gait of a subject with Guillain–Barré syndrome before and after being provided with information regarding other movements. Further, visual lines were measured using an EMR-8 eye mark recorder, and the results were compared between both groups. The average gaze duration was longer for students than for PTs (F1,79=53.3; p<0.01), whereas PTs gazed more often than the students (F1,79=87.6; p< 0.01). Furthermore, the PTs moved their eyes vertically more often than the students (F1,151=9.1; P< 0.01). We found that being able to discriminate the relative physical relationship of body locations by frequent and rapid vertical gazes could be an indication of the level of skills as an index to express the visual assessment skill in an observation-based gait analysis.

## Introduction

Gait, or human walking, is a significant predictor of quality of life, morbidity, and mortality (Hulleck et al, 2022).　Gait patterns and other kinematic, kinetic, and balance gait features are accurate and powerful diagnostic and prognostic tools. Quantitative Instrumented gait analysis (IGA) has the capability of providing clinicians with accurate and reliable gait data for diagnosis and monitoring but is limited in clinical applicability mainly due to logistics (Katmah R et al, 2023).

The physiotherapist (PT) can immediately determine whether the patient has an abnormal or healthy gait through observational gait analysis. Furthermore, in the case of abnormal gait, it is possible to narrow down the location of joint and muscle abnormalities. The Observational Gait Instructor Group has systematized observational gait analysis, a qualitative approach to gait analysis used by clinicians, in which gait deviations in patients can be visualized. Previous studies on observational gait analysis have primarily focused on accuracy and reliability. Krebs et al evaluated the gait patterns in 15 children with lower limb disabilities requiring knee–ankle–foot orthosis and found poor reliability of the retest observations for gait parameters (12). Saleh et al found that clinicians observing prosthetic alignment detected only 22% of the deviations predicted by the biomechanical gait analysis (15). Miyazaki et al reported a mean Pearson’s product moment correlation (r) of 0.55 between observations of selected gait components and waveform indexes using a device to measure the foot force (13). These findings indicated that gait analysis by visual observation is only moderately reliable and accurate (4. 10. 3).

Few studies have shown correlations between clinical experience and reliability during visual gait analysis, although many others have indicated that experience does not influence the visual gait analysis. However, visual lines may be affected by an observer’s experience. A study on the reliability of measurements identifying angular joint movements compared with that of observations found that the effect of experience was not dependent on the ability of an observer to discriminate between joint movements but rather on the ability to recognize and judge them as deviant and identify potential causes (2). This is because those who closely observe specific movements during gait analysis move their eyes.

Eye movements can indicate where others direct their gaze and focus their attention, but they cannot indicate whether an item is stored into memory for analysis. Akiko N et al measured eye movements with an Eye Tracker and prefrontal cortex activity using a wearable Optical Topography in 18 participants performing a visual working memory task. As a result, it revealed that increased brain activity and higher fixation counts were related to improved task performance (1).

In Studies in Eye Movement Behavior in Novice and Experienced Billiard Players, by testing different combinations of simple oculomotor features (gaze shifts amplitude and direction, and fixation duration), we could classify on an individual basis which group - novice or expert - the observers belonged to with an accuracy of 82% and 87%, respectively for the match and the shots. The result provides evidence that a signature of expertise is hidden in very basic aspects of oculomotor behaviour (9).

Thus, from the characteristics of eye movements, it is possible to extrapolate important information about expertise in several knowledge and activity domains. Eye movements can be a useful source of information for inferring cognitive processes. Observational gait analysis is a clinical inference by narrowing down the problem from the patient's gait. The observer's eye movements are considered indicative of this cognitive process.

This study aimed to (1) compare eye movements between experienced physiotherapists (PTs) and students and (2) identify characteristics of observational skills by analyzing the eye movement in an observation-based gait analysis.

## Methods

To determine the skill level involved in an observation-based gait analysis, we compared visual lines during gait observations between PTs with >10 years of experience and students.

### Participants

Observers; We included 20 PTs (39.9±7.0 age) with >10 years of clinical experience (PTs)and 20 fourth-year physiotherapy students (25.1±2.7 age) in this study. Students underwent clinical training at a hospital and visually analyzed gait daily for 8 weeks during their fourth year.

Subject of observation: The gait of a 73-year-old man (height, 160 cm; weight, 67 kg) with Guillain–Barré syndrome, muscle weakness, and peripheral nerve disease who could walk without crutches was analyzed. The results of a manual muscle test indicated level P in bilateral ankle plantar flexion, level F–G in knee and hip extension, and the patient’s right side was stronger than his left. The range of motion analysis indicated no specific issues.

### Design

Both groups watched a video of the gait of a subject with Guillain–Barré syndrome before and after being provided with information regarding other movements. Further, visual lines were measured using an EMR-8 eye mark recorder, and the results were compared between both groups.

The gaze duration and number, how often the gaze shifts to search for a location on the subject’s body, and the location that received the most focus were analyzed in a frame-by-frame analysis. We also assessed the differences in each item between the groups, changes in values after presenting the participants with information that may affect the subject’s gait, and the sagittal and coronal planes of motion.

### Materials

The subject was videotaped while walking outdoors and indoors at a comfortable speed. The coronal and sagittal planes of motion were recorded using video cameras positioned near the middle and at the end of a 10-m walkway. The coronal plane camera was positioned 2 m from the middle of the walkway with a self-focusing lens that allowed for coronal plane evaluation along the walkway length. The sagittal plane camera was positioned near the middle and 3 m to the side of the walkway. This arrangement allowed evaluation of one complete stride as the subject passed by the camera during each 10-m walk. Both cameras were aligned parallel to the ground and positioned such that the image captured by each was centered on the subject’s body while on the walkway.

The tape was edited to a duration of 5 min and 40 s. The outdoor and indoor gait was analyzed for 1 min and 50 s. After creating a series of gait videos, the PTs and students received a 2-min presentation with information associated with the subject’s movements (i.e., foot movement, half kneeling, and standing on one leg). Thereafter, the video series of the subject’s gait was observed again.

In addition, the subject’s height appeared reduced on the screen; therefore, we excluded the data when the height of the subject on the screen was one-third or less than the subject’s actual height.

The visual line during the observation was determined using an eye mark recorder with a lens angle of view of 92° and a mini digital video camcorder. The PTs and students visually analyzed a video of the subject’s gait on a 15-inch screen while seated and wearing a head unit. Head movement could be suppressed using the elbow, and the chin was fixed.

### Procedure

The video was initially viewed once by the PTs and students while blinded to any information regarding the disease or causes of gait deviation and then once again after being provided with information about factors that may have affected the subject’s gait. The visual line was analyzed by a frame-by-frame analysis of a video and the eye mark recorder (5). A video from the eye mark recorder was transferred to a computer and then the results of the frame-by-frame analysis were expressed as gaze points on the images using Microsoft Excel.

**Figure 1.**
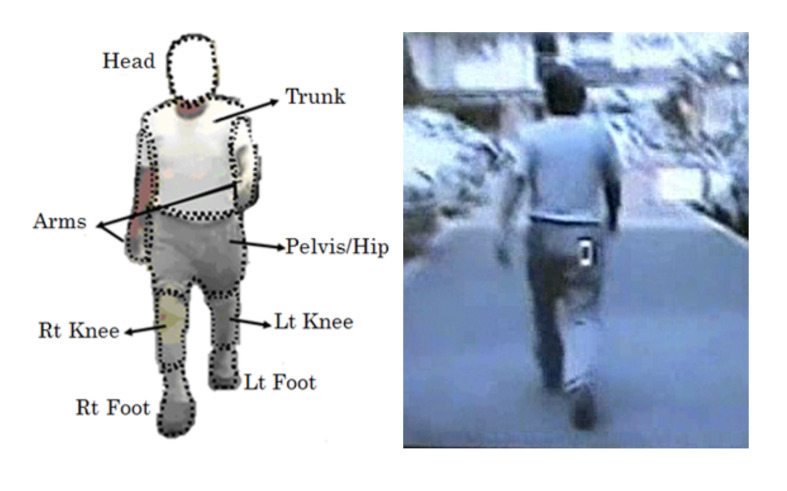
Locations marked on body of observed subject.

**Figure 2.**
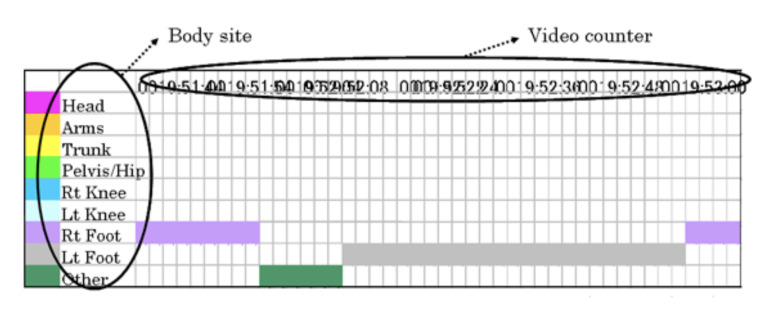
Visual line analysis (Microsoft Excel). Video counters are shown in rows and the location on the body is shown in columns (Fig. 1). From the information provided by the eye mark recorder, lines were matched in Microsoft Excel using the frame counter. Definition of the gaze duration, held for 0.12 s for >4 frames.

We marked some locations on the subject to spatially define the eye placement for the frame-by-frame analysis (Fig. 1). From the information provided by the eye mark recorder, the lines were matched using the frame counter in the Microsoft Excel program, i.e., a line was engraved every 0.03 s and counted as one frame. The vertical line was assumed to represent a marked location on the subject’s body. A frame-by-frame analysis list was prepared (Fig. 2). Ten frames were generated from each observer while observing the subject’s gait in 10 scenes before and after receiving information regarding the causes of the subject’s gait deviation resulting in an analysis list of 20 frames.

The analyzed items were gaze duration and number, the location on the body where the gaze was focused, and the number of eye movements involved while searching for a location upon which to gaze (6, 7).

Gaze duration was defined as the amount of time the eyes remained fixed on one marked location, and the number of gaze frames was counted in each scene. We defined gaze duration as >0.12 s as described by Fukuda and assumed that the eyes remaining fixed on the same body location for more than four frames that comprised a gaze state (5). Data were extracted from at least three frames. The number of gazes fixed on each marked body location was counted, and the gaze duration fixed on each location was expressed as a ratio to the total gaze duration. The number of times the observer’s eyes moved in search of a location upon which to gaze was expressed as a number. Data collection began when the video started. We assumed one step was from the left heel contact to the next left heel contact in one frame, and six steps were counted.

Values obtained before and after the observers were provided with information about factors affecting the subject’s gait were analyzed using a two-way analysis of variance (ANOVA) and a multiple comparison test after confirming normal distribution. If the ANOVA indicated a significant difference, mean values were assessed using the Tukey–Kramer multiple comparison procedure. Correlation coefficients were calculated between pairs of the analyzed items. All data were analyzed using StatView J–5.0 software. A p-value>0.05 was considered statistically significant.

**Figure 3.**
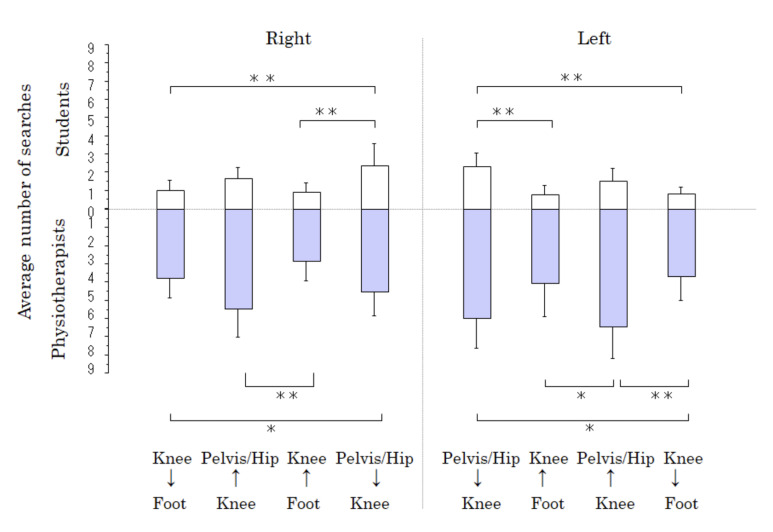
The average number of shifts during the vertical gaze to observe marked locations. White bar, students. Gray bar, physiotherapists. *p < 0.05; **p < 0.01.

## Results

The average gaze duration and number were determined before and after providing the PTs and students with information regarding factors that may affect the subject’s gait (Table 1). The average gaze duration was longer for students than for PTs (F1,79=53.3; p <0.01), whereas the PTs gazed more often than the students (F1,79=87.6; p<0.01). Providing the two groups with information regarding the subject’s gait did not significantly affect the outcomes.

The gaze was directed significantly more often towards the pelvis and hips (p<0.01) by both groups, and students more often gazed at all locations (p < 0.05).

Table 2 shows the average number of gaze shifts to find a location on the subject’s body before and after being provided with information. The PTs significantly more often shifted their gaze vertically in search of a location than students before and after being provided with information (F1,159=23.7; p< 0.01). The numbers of vertical and horizontal gaze shifts were similar among the PTs, whereas the students gazed horizontally more often than vertically (p<0.01). After providing information, the numbers of vertical gaze shifts did not significantly differ among the PTs.

Figure 3 shows the average number of gaze shifts to find a location on the subject’s body. The PTs and students significantly more often shifted their gaze vertically from the pelvis and hips to the knees than from the knees to the foot (p<0.05 and p<0.01, respectively). In addition, the PTs significantly more often shifted their gaze vertically from the knee to the pelvis and hip than from the foot to bilateral knees (p<0.05 and p<0.01, respectively).

As shown in Table 3, significant differences were observed between the average gaze duration and the average number of gazes between the two groups (r=−0.50 and −0.87; p < 0.05). The average gaze duration negatively correlated with the average number of vertical gaze shifts to find a location (r=−0.45; p <0.05) among the PTs but not among the students. A negative correlation was observed between the average gaze duration and the average number of horizontal gaze shifts to find a target (r=−0.62; p<0.01) among the students but not among the PTs. Both groups showed a positive coefficient for the average horizontal gaze duration, and a part of the gaze duration was spent on the subject’s knees and feet (r = 0.45 and 0.71; p < 0.05).

**Table 1.**
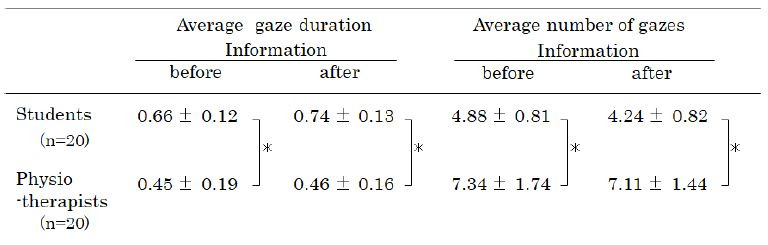
Changes in average gaze duration and number of gazes after providing the observers with information about the gait of the subject

Note. Values are presented as the means ± SD *indicates the analysis of variance (ANOVA) results (p < 0.01) within each group

**Table 2.**
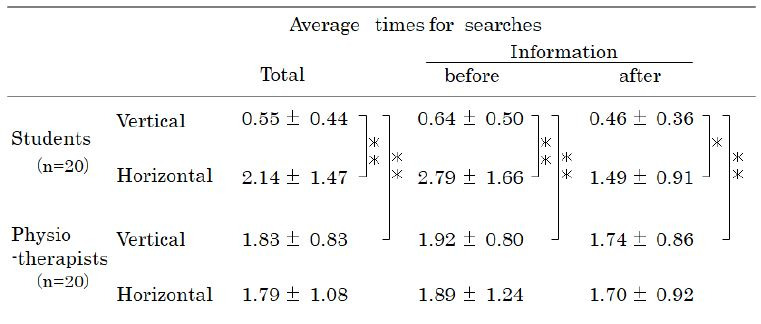
The average number of gaze shifts to find a location on the subject’s body before and after being provided with information

Values are presented as the means ± SDs *p < 0.05; **p < 0.01

**Table 3.**
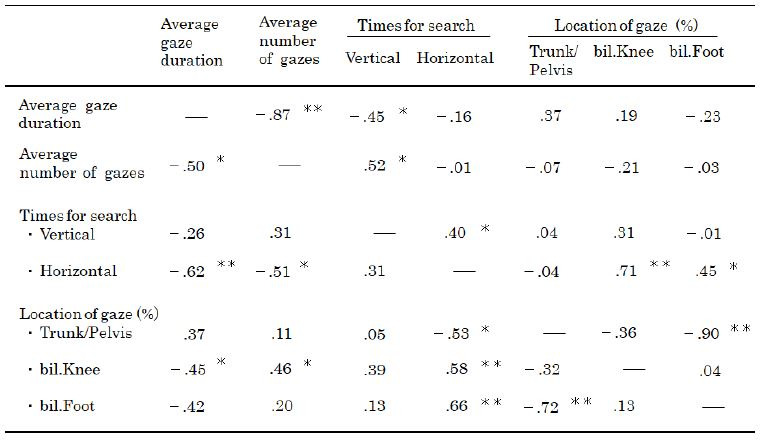
The correlation coefficients for analysis of items by the students and physiotherapists

bil, bilateral. Values are presented as correlation coefficients. Lower left, students; upper right, physiotherapists *p < 0.05; **p < 0.01

## Discussion

To determine the skill level involved in an observation-based gait analysis, we compared visual lines during gait observations between PTs with >10 years of experience and students. The gaze duration and number, how often the gaze shifts to search for a location on the subject’s body, and the location that received the most focus were analyzed in a frame-by-frame analysis. We also assessed the differences in each item between the groups, changes in values after presenting the participants with information that may affect the subject’s gait, and the sagittal and coronal planes of motion.

The average gaze duration among the PTs was shorter and they gazed more frequently than the students. These findings indicated that the PTs consistently moved their visual lines without spending much time gazing at a single target. One gait cycle of the videotaped subject took an average of about 1.3 s. The students recognized two body parts in one gait cycle, whereas the PTs recognized an average of three. These findings indicated that recognizing the relative movement of each location shortened the gaze duration and allowed more frequent eye movement. Yoshida et al investigated the visual lines in four PTs and four students using an eye mark recorder and a video during observation of a hemiplegic patient, but no apparent difference was found in the average gaze duration between the two groups (16). Because the average gaze duration and the number of gazes were determined by the number of locations on the subject’s body at which an observer closely gazes during a gait cycle, these parameters may be affected by the nature of the disease and the gait speed of the videotaped subject as well as the quantity and nature of information provided to the observers.

The PTs gazed more often at the pelvis and hip joint than at any other body part. Ford at al found that experts in general and PTs in particular also observed the head, arms, and trunk rather than only the lower limbs (8). In the present study, only the pelvis of the videotaped subject could be defined. However, the gaze location may be clear if an expert observer gazed in a particular manner based on the disease state.

The PTs significantly more often used their vertical gaze to search for locations than the students, whereas the students used their horizontal gaze more often than their vertical gaze. Furthermore, the average gaze duration and the average number of vertical gazes negatively correlated among the PTs but not among the students. This finding suggested that the students laterally distinguished each location, unlike the PTs who distinguished the relevance of each location by vertically and horizontally moving their eyes. The PTs searched for locations at which to gaze from the foot to the knee and hip and from the proximal to the distal side by shortening the gaze duration. We propose that the PTs can differentiate among the relative physical relationships of each body location. Because the observed subject in the present study suffered from paralysis due to the peripheral neuropathy, the observer’s eyes may have moved to distinguish vicarious from abnormal movements of the foot to the knee, pelvis, and hip. Thus, the observer’s eyes may move to predict changes in each gait phase. Therefore, we propose that vertical searching is a factor representative of the degree of skill in an observational gait analysis.

The number of searches for a location at which to gaze after providing the PTs with information about the observed subject did not differ, but the number of horizontal gazes decreased after providing the same information to the students. We predicted that acquiring information about the disability, disease, and laterality of the muscle strength will change the gaze movement in an observational gait analysis, but this was not true for the PTs whose observation patterns were stable. Furthermore, we speculated that the PTs had already narrowed down potential problems by shortening their gaze duration and increasing the number of gazes in the 10 scenes before receiving information about the subject.

According to Read et al coronal plane observations are the most difficult to interpret (14). In general, the body height can be confirmed from the right and left sides by actual measurements; thus, we predicted that the number of horizontal gazes may increase. The PTs significantly more often gazed vertically than horizontally. However, we were unable to assess searching eye movements in the coronal plane because we did not divide the right and left sides of the trunk and pelvis when we established the gaze locations.

In this study, only one subject with bilateral peripheral neuropathy was observed. A characteristic of the special visual lines caused by the obstacle may appear, thereby limiting the generalization of these results as the skill of the observer. In addition, it was impossible to clarify how observations recognized problems by only using visual lines.

In the future, the number of diseases and movement disorders to be observed will be increased and the experiential skills of gait analysis by observation will be clarified.

### Ethics and Conflict of Interest

The author(s) declare(s) that the contents of the article are in agreement with the ethics described in http://biblio.unibe.ch/portale/elibrary/BOP/jemr/ethics.html and that there is no conflict of interest regarding the publication of this paper.

### Acknowledgements

This work was supported by the University of Health and Welfare Graduate School. The study sponsors had no involvement in the design of the experiment, the collection, analysis, or interpretation of the data, or in the writing or submission of this manuscript.
